# Peripheral Nerve Regeneration Reimagined: Cutting-Edge Biomaterials and Biotechnological Innovations

**DOI:** 10.3390/bioengineering12080864

**Published:** 2025-08-11

**Authors:** Ting Chak Lam, Zhenzhen Wu, Sang Jin Lee, Yiu Yan Leung

**Affiliations:** 1 Department of Oral and Maxillofacial Surgery, Faculty of Dentistry, University of Hong Kong, Hong Kong; u3507413@connect.hku.hk; 2 Department of Applied Oral Sciences and Community Dental Care, Faculty of Dentistry, University of Hong Kong, Hong Kong; wuzzwork@connect.hku.hk (Z.W.); dentsj@hku.hk (S.J.L.)

**Keywords:** nerve regeneration, nerve guidance conduits, peripheral nerve injury

## Abstract

Peripheral nerve injuries are frequent clinical issues that can lead to significant functional impairments, greatly impacting patients’ quality of life. Developing effective nerve regeneration methods is crucial for restoring function and ensuring the best possible outcomes. This review explores recent advances in nerve regeneration, including nerve guidance conduits (NGCs), which are vital in bridging nerve gaps caused by injury and supporting repair. The field has seen significant progress in biomaterials and biotech, with biodegradable options like collagen and chitosan as well as non-biodegradable materials such as nylon. Innovations like 3D printing have allowed for more intricate conduit designs that more closely mimic natural nerves. Despite these progressions, research continues to focus on improving NGCs—often by adding cells or bioactive substances—to boost nerve regeneration and functional recovery. By analyzing current trends, this review aims to motivate clinicians and researchers to develop more comprehensive nerve repair strategies. It emphasizes approaches that combine scientific innovation with clinical practicality, fostering a more holistic and realistic outlook on enhancing patient outcomes in peripheral nerve regeneration.

## 1. Introduction

Peripheral nerve damage poses a substantial challenge for healthcare practitioners, potentially causing mobility and sensory deficits, chronic pain, and a reduced quality of life [1]. In the United States, the incidence of peripheral nerve damage ranges from 10 to 20 cases per 100,000 individuals annually [2]. Surgical intervention is often necessary for direct nerve repair, which may involve nerve transplantation or transfer. Direct nerve repair is the preferred method when the nerve ends can be aligned without significant tension, achievable only if the injury is not severe and if the gap between the nerve ends is small [3]. On the other hand, nerve grafting is frequently used to bridge larger gaps, aiding in nerve regeneration [4]. Autologous nerve grafts remain the most effective means of closing nerve gaps. Nevertheless, challenges remain, such as donor site morbidity, limited availability of donor nerves, and the risk of further nerve damage to the patient [5]. Despite improvements in surgical techniques, achieving complete recovery after nerve repair is still difficult [6]. Factors including delays in treatment, the severity of the injury, and the patient’s age can complicate and hinder healing processes. Incorrect connections of regenerated axons can worsen healing outcomes, resulting in functional impairments [7] and potential muscle atrophy from inactivity, which further reduces muscle functionality.

Current research is primarily centered on bioengineering materials and technologies designed to improve neuronal repair after injury [8]. Nerve guidance conduits present a promising alternative to traditional nerve grafting techniques for addressing nerve injuries, particularly when tensionless neurorrhaphy is not feasible. These conduits are typically recommended when the disconnection gap surpasses the natural capacity of peripheral nerves. Researchers have been investigating a diverse range of materials, including both synthetic and natural polymers as well as composite materials, for creating nerve-guiding conduits or scaffolds that facilitate nerve repair and regeneration. The addition of bioactive agents like growth factors into these conduits is expected to boost their efficacy in supporting nerve healing [9]. Cutting-edge technologies such as 3D printing and nanotechnology have allowed for the integration of complex designs in the conduits and controlled drug release mechanisms [10].

**Figure 1:**
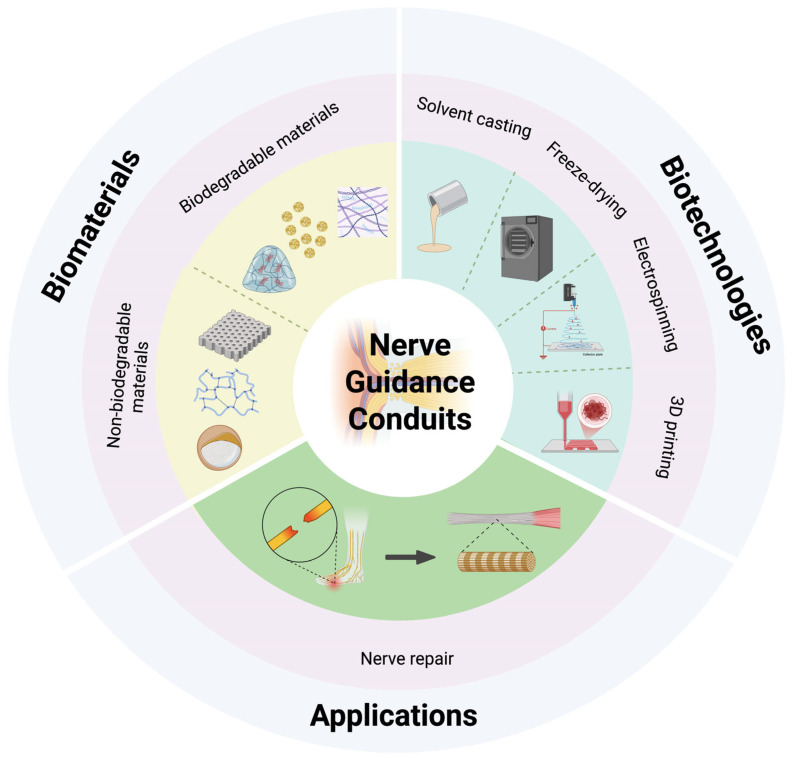
Schematic illustration of representative nerve guidance conduit designs (created in https://BioRender.com).

This study explores and organizes the latest biomaterials and biotechnologies for repairing injured peripheral nerves (Figure 1). It examines modern engineering techniques that can be employed to produce neural conduits with targeted properties.

## 2. Advanced Biomaterials for Peripheral Nerve Regeneration

### 2.1. Biodegradable Materials

#### 2.1.1. Collagen

Collagen, a crucial element of the extracellular matrix, has emerged as a vital biomaterial in nerve repair and regeneration due to its excellent biocompatibility, biodegradability, and ability to promote cellular adhesion and growth. Its structural features facilitate neurite outgrowth and revascularization, which are advantageous for developing NGCs [11]. However, collagen encounters certain obstacles, including limited mechanical strength and susceptibility to enzymatic degradation, which can diminish its long-term effectiveness [12]. To address these limitations, recent initiatives have focused on modifying the surfaces of collagen scaffolds and incorporating bioactive components like growth factors and stem cells to boost their regenerative capabilities [13]. By enhancing these collagen-based systems, researchers intend to improve the outcomes of peripheral nerve injuries, leading to better clinical treatments [14].

**Figure 2:**
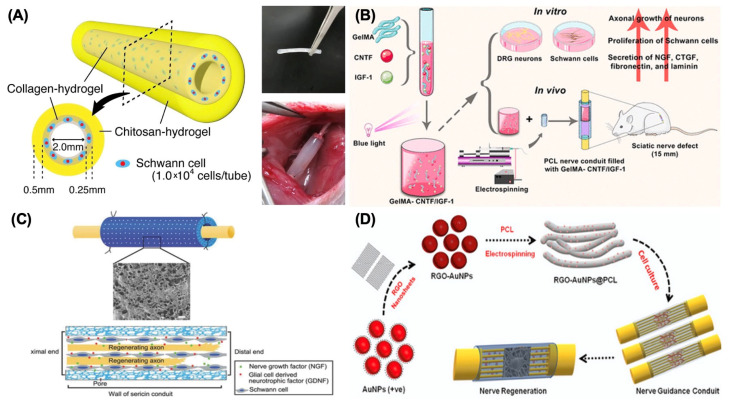
Different kinds of biomaterials for NGCs. (**A**) Design of chitosan–collagen hydrogel nerve guidance conduit. Adapted with permission from Takeya et al. [19] Copyright (2023), Springer. (**B**) Design of electrospun polycaprolactone (PCL) nerve conduit incorporating methacrylate-anhydride gelatin (GelMA) for the delivery of ciliary neurotrophic factor (CNTF) and insulin-like growth factor-1 (IGF-1) to enhance sciatic nerve regeneration. Adapted with permission from Xu et al. [20] Copyright (2023), American Chemical Society. (**C**) Design of fabricating a sericin/silicone nerve guidance conduit. Adapted with permission from Xie et al. [21] Copyright (2015), Wiley. (**D**) Schematic illustration for the synthesis of monodispersed plasmonic gold nanoparticles (AuNPs)–reduced graphene oxide (rGO)-integrated polycaprolactone (PCL) composite nanofiber electroconductive smart scaffold for nerve regeneration. Adapted with permission from Jaswal et al. [22] Copyright (2023), Elsevier.

In the past five years, research on collagen is concentrating on developing more sophisticated designs and architectures for conduits. New conduit structures have emerged, such as microchannels designed for precise chemical delivery of neurotrophic factors [15] and collagen filaments added to collagen-based conduits to improve their structural integrity [16]. To resolve potential anatomical mismatches between conduits and nerve stumps, 3D-printed collagen-based conduits have been created to enhance the efficiency of nerve repair and regeneration by promoting better integration with the patient’s biological systems [17]. In addition to architectural improvements, the inclusion of bioactive substances presents another pathway for development. Table 1 illustrates the summary of recent research on collagen as a biomaterial for nerve regeneration. For instance, Kim et al. [18] combined platelet-rich plasma (PRP) with collagen in conduits, demonstrating enhanced nerve regeneration, as PRP fosters collagen synthesis and neuronal growth. This study revealed that different doses of platelet-rich plasma significantly increased the levels of neurotrophic factors and myelin-associated glycoprotein, which supports functional recovery. Researchers are also exploring composite materials to address the biomechanical shortcomings of collagen. For example, Takeya et al. [19] assessed a novel hydrogel nerve conduit featuring a core-shell nanostructure, with an outer layer of chitosan hydrogel and an inner layer of collagen hydrogel enclosing Schwann cells (Figure 2A). Their results indicated that Schwann cell encapsulation substantially enhanced peripheral nerve regeneration, particularly axonal regrowth and remyelination.

#### 2.1.2. Chitosan

Chitosan, a biopolymer derived from chitin in crustacean exoskeletons, has attracted considerable interest as a biomaterial for nerve repair and regeneration. Its distinctive features, such as biodegradability, biocompatibility, and natural antimicrobial properties, make chitosan an exceptional candidate for NGCs. A key advantage of chitosan is its capability to promote Schwann cell adhesion and migration, which are vital for successful nerve regeneration. Additionally, the degradation product of chitosan, chito-oligosaccharide (COS), has been demonstrated to stimulate cell proliferation, thereby aiding in the regeneration process. Chitosan exhibits a lower Young’s modulus than both collagen and peripheral nerve tissue, offering reduced resistance to stretching while preserving the tensile strength typical of nerves. This characteristic not only minimizes the risk of failure during manufacturing but also enhances Schwann cell migration and supports axonal regeneration at the reconstruction site during movement. Nevertheless, concerns regarding its mechanical strength and structural stability in physiological conditions remain, potentially limiting its effectiveness across longer nerve gaps. It is essential to understand these benefits and limitations as researchers strive to improve chitosan-based solutions for peripheral nerve injuries.

Table 2 illustrates the overview of recent research on chitosan as a biomaterial for nerve regeneration. Deng et al. [23] introduced a chitosan-based NGC featuring a multifunctional bilayer structure designed to enhance mechanical stability while enabling controlled pharmaceutical agent release, which significantly increased MBP expression levels-comparable to those of autologous tissue. Furthermore, the morphological diversity of chitosan presents additional development opportunities. Chitosan microspheres infused with adipose-derived stem cells have been proposed by Zhu et al. [15] to establish a porous framework that enhances cellular movement and retention at injury sites, leading to improved nerve regeneration in a 12 mm sciatic nerve injury model in rats. Another porous model introduced by Li et al. [24] involved a lysine-modified chitosan scaffold that was injected with Matrilin-2, promoting Schwann cell migration and axonal growth both in vitro and in vivo.

#### 2.1.3. Gelatin Methacryloyl (GelMA)

Gelatin methacryloyl (GelMA) is an innovative biomaterial that has been extensively researched for its potential in nerve repair and regeneration due to its unique tunable properties. GelMA is a modified gelatin that enables photocrosslinking, allowing for the creation of hydrogels with adjustable mechanical strengths and degradation rates, which are ideal for various tissue engineering applications. Its biocompatibility and ability to promote cell adhesion significantly enhance Schwann cell proliferation and migration, both of which are critical for nerve regeneration. However, GelMA also encounters challenges as a biomaterial, such as lower mechanical strength compared to conventional materials used in nerve conduits and a tendency for rapid degradation, which could limit its effectiveness in scenarios requiring long-term support. It is vital to balance these advantages and drawbacks to optimize GelMA for nerve guidance conduits and improve peripheral nerve repair methods.

Similar to the research work with collagen, scientists are also committed to incorporating growth factors into GelMA-based hydrogels to enhance functional recovery. Table 3 concludes the recent research on GelMA as a biomaterial for nerve regeneration. For example, Cai et al. developed a nerve guidance conduit infused with graphene by combining a natural double network hydrogel with silk fibroin and GelMA, which included a neurotrophic concentration gradient in a diabetic neuropathy model [25]. This intricate model reportedly provided adequate mechanical support and consistent delivery of growth factors. In addition, polycaprolactone was used to create a composite conduit with GelMA, improving structural integrity and biocompatibility [20,26]. One study involved electrospun polycaprolactone (PCL) conduits infused with neurotrophic growth factors, developed by Xu et al. [20], resulting in better functional recovery in a rat sciatic nerve injury model. Specifically, they integrated the properties of GelMA with MMP enzymatic degradation to develop MMP-responsive hydrogels that gradually release ciliary neurotrophic factor (CNTF) and insulin-like growth factor-1 (IGF-1), with reports indicating enhanced myelination, axonal regeneration, and angiogenesis (Figure 2B).

#### 2.1.4. Biodegradable Synthetic Polymers

Biodegradable synthetic materials such as polycaprolactone (PCL), poly(lactic-co-glycolic acid) (PLGA), and poly(lactic acid) (PLA) are becoming increasingly popular for nerve repair and regeneration, and it is easy to see why. Each of these materials possesses unique features that make them especially suitable for this purpose. For example, PCL is renowned for its excellent mechanical strength and biocompatibility, making it a robust choice for constructing nerve conduits [27]. PLGA is notable for its adjustable degradation rates and FDA approval, which ensure its compatibility with the body [28]. Although PLA is also frequently used, it has some disadvantages, such as water absorption and lower cell affinity, which can complicate its application in tissue engineering [29]. One of the main advantages of these materials is their biodegradability, which helps address ongoing concerns about the permanence of implants in the body. They are designed not only to remain in place but also to promote cell attachment and, at least temporarily, restore nerve function [30,31]. However, they also face challenges. Issues such as limited mechanical strength, potential inflammation, and varying degradation rates can impact their effectiveness in supporting nerve regeneration [32,33].

Exciting progress has recently been made in using biodegradable synthetic materials for nerve repair and Table 4 demonstrates some recent research on biodegradable synthetic polymers for nerve regeneration. For instance, research conducted by Kusuhara et al. [34] has shown that combining polyglycolic acid (PGA) with collagen scaffolding yields remarkable results, particularly in supporting sensory recovery while reducing inflammation in bridging nerve gaps. In another study, Cicero et al. [35] found that poly-butylene succinate (PBS) scaffolds are effective in promoting nerve regeneration in living organisms, demonstrating both biocompatibility and decreased inflammation. There have even been advancements involving PLGA nanomaterials infused with specific nutrients, which have successfully been used to address challenging long-distance peripheral nerve injuries, leading to improved structural and functional recovery [28]. Furthermore, blending biodegradable synthetic materials with other compounds or modifying their surfaces is proving to be a promising approach to encourage nerve regeneration. For instance, Nazeri et al. [36] illustrated that adding laminin to the surfaces of PLGA scaffolds can significantly enhance neurite growth and improve cell interactions—an important modification that shows great potential. Similarly, multi-channel electro-conductive conduits made from PCL and PLGA were developed by the same research group, demonstrating potential for stimulating cell growth and improving nerve healing [37]. Furthermore, incorporating neurotrophic factors into these conduits has also shown that such modifications can greatly enhance their regenerative capabilities [38].

### 2.2. Non-Biodegradable Materials

#### 2.2.1. Polytetrafluorothylene

Polytetrafluoroethylene (PTFE) is a synthetic fluoropolymer renowned for its exceptional chemical and thermal stability as well as outstanding biocompatibility. These characteristics make it a preferred material for various biomedical uses, especially because it can be sterilized without inducing adverse physiological responses. As a result, PTFE is an excellent candidate for nerve regeneration scaffolds. It has been widely studied as a focal material in nerve regeneration research and the development of nerve conduits. However, PTFE lacks inherent biological activity, necessitating modifications to enhance its interaction with neural tissues and accelerate nerve repair [39].

Recent developments in PTFE research for nerve regeneration emphasize the importance of combining PTFE with other bioactive materials or drug delivery systems along with targeted surface modifications. Table 5 concludes recent research on PTFE as a biomaterial for nerve regeneration and the research results indicate that PTFE-based nerve-guiding conduits have notably improved regeneration outcomes. Labroo et al. [40] studied PTFE conduits infused with glial cell line-derived neurotrophic factor (GDNF) and FK 506. They also utilized micro-grooved surfaces and porous features that enable the controlled release of bioactive substances, thus guiding nerve fibers and facilitating cell entry. Such modifications have been shown to enhance the neural regeneration environment by supporting neuronal cell adhesion and encouraging robust axon growth through the conduit. This study demonstrated that a hole-based drug delivery system efficiently releases bioactive growth factor concentrations at a controlled pace, thereby promoting nerve growth. Additionally, combining PTFE with polyethene glycol (PEG) and collagen has been proposed to decrease PTFE’s hydrophobicity by Kahraman et al. [41], thus improving cellular adhesion and biocompatibility. Furthermore, functional recovery and nerve regeneration were significantly greater than with primary repair, with no significant changes in epineural and extraneural scar tissue formation.

#### 2.2.2. Silicone

Silicone has long been recognized as a crucial biomaterial for nerve repair and regeneration, particularly in the context of NGCs. Its excellent biocompatibility, chemical stability, and flexibility facilitate the growth of regenerating nerve fibers while acting as a protective barrier against scar formation. Due to its ability to remain implanted without eliciting negative reactions, silicone has become a standard in clinical settings. However, there are limitations, including insufficient bioactivity and a tendency to cause fibrous encapsulation, which can impede nerve regeneration. Furthermore, silicone conduits may not deliver the same level of functional recovery as alternative materials or methods that incorporate cell- or matrix-based enhancements. As a result, although silicone is a viable option for NGCs, addressing its shortcomings through innovative approaches is vital for improving outcomes in peripheral nerve repair.

To enhance silicone’s effectiveness in nerve repair, combining it with natural biomaterials like silk has shown promising results. Table 6 illustrates the summary of recent research on silicone as a biomaterial for nerve regeneration. For instance, a study conducted by Xie et al. [21] revealed that a silk sericin/silicone nerve guidance conduit not only offered a supportive structure for nerve repair but also facilitated better regeneration of transfected sciatic nerves in animal models (Figure 2C). This combination leverages the biocompatibility of silk sericin with the mechanical strength of silicone, resulting in improved muscle weight recovery and nerve regeneration compared to silicone conduits alone. Additionally, silicone conduits have been successfully integrated with advanced drug delivery systems. For example, the addition of growth factors, such as nerve growth factor (NGF), into silicone conduits enables localized release, thereby enhancing the regenerative conditions for damaged nerves. Velichanskaya et al. [42] employed a silicone conduit infused with a mixture of adipose-derived stem cells (ADSCs) and collagen to enhance nerve regeneration. Their study reported promising levels of regeneration, emphasizing the benefits of combining silicone with live cell therapies.

#### 2.2.3. Smart Polymers

Smart polymers represent a cutting-edge category of biomaterials that respond to environmental factors such as temperature and pH. Their ability to respond to these changes makes them particularly appealing for nerve repair and regeneration. These polymers can alter their physical and chemical properties in response to environmental variations, facilitating controlled drug release, enhanced cellular interactions, and improved compatibility with nerve tissues. A key advantage of smart polymers is their ability to dynamically support regenerating nerves, contributing to an environment that fosters axonal growth and repair. However, challenges exist, such as potential mechanical instability under varying conditions and the complexities involved in their synthesis and processing. There is a need for additional efforts to improve the functionality of these materials and their integration into clinical applications, ensuring they effectively act as nerve guidance conduits for peripheral nerve regeneration.

Recent studies have explored the combination of electrical stimulation with smart polymers to significantly improve nerve regeneration outcomes, as nerve cells exhibit a robust regenerative response to electrical stimulation (ES) and Table 7 concludes some recent research on smart polymers as biomaterials for nerve regeneration. For example, Rahman et al. [43] observed that incorporating electrically conductive biomaterials into the development of smart NGCs amplifies the effects of electrical stimulation during the active proliferation phase. This synergistic approach has demonstrated enhancements in both functional recovery and histological assessments of nerve regeneration across different animal models. Furthermore, these smart polymer-based conduits can be 3D-printed to meet individual patient needs. By integrating growth factors and cells into the bioactive inks, it is possible to create a supportive healing environment through controlled release mechanisms. Three-dimensionally printed smart polymer conduits not only improve structural properties by maximizing the biochemical support essential for nerve regeneration but also create a responsive environment that adapts to physiological changes, facilitating nerve healing. For instance, thermo-responsive shape memory polymers (SMPs) like poly(lactide-co-trimethylene carbonate) are capable of maintaining a stable tube structure despite temperature variations in the body [44]. In addition to these materials, which produce composite conduits with intricate designs, researchers are investigating the use of smart polymers and nanoparticles to enhance healing effectiveness and expand the functionality of nerve conduits. Jaswal et al. [22] developed the nano-sized reduced graphene oxide (RGO)-enfolded gold nanoparticles (AuNPs) and combined the produced AuNPs@RGO with PCL by electrospinning, as shown in Figure 2D, emphasizing how the inclusion of conductive nanoparticles can significantly bolster the mechanical and electrical properties of smart polymers, thereby enhancing the conduit’s ability to support axonal growth and guidance.

## 3. Fabrication Strategies for Peripheral Nerve Regeneration

### 3.1. Solvent Casting

**Figure 3:**
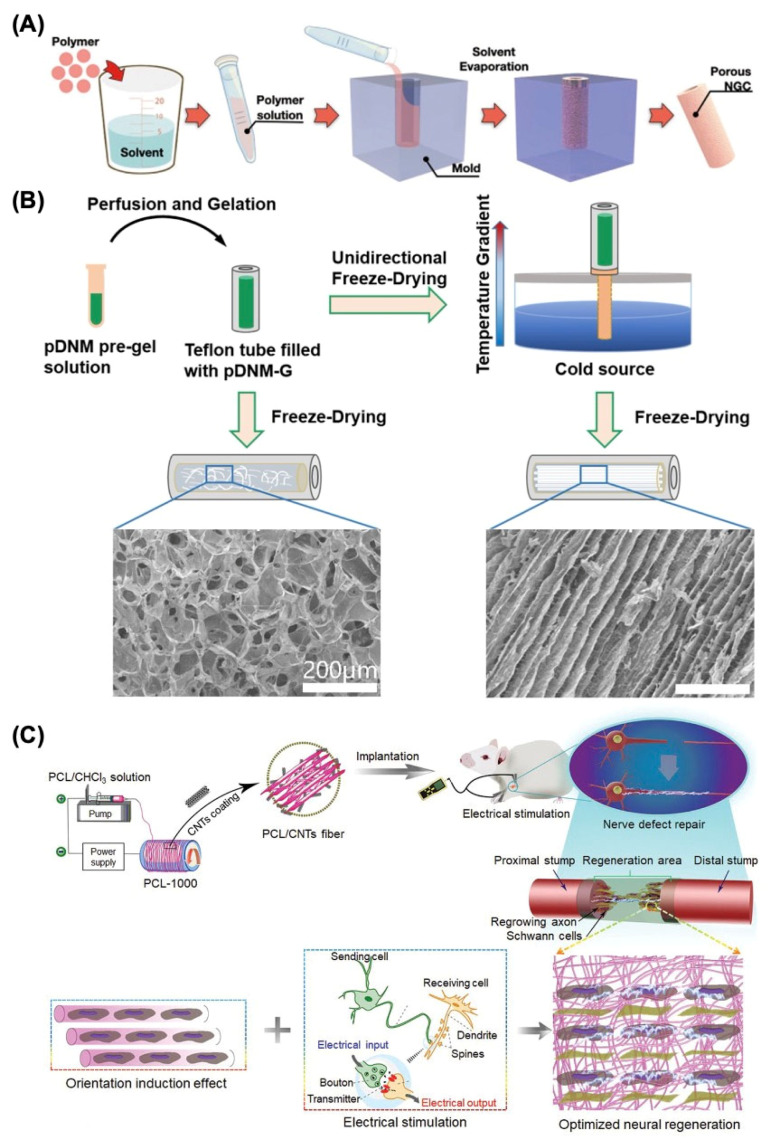
Traditional fabrication methods for nerve guide conduit construction. (**A**) Solvent casting. Adapted with permission from Kang et al. [20] Copyright (2022) Yonsei University College of Medicine. (**B**) Freeze-drying process for producing a decellularized nerve matrix hydrogel derived from porcine sciatic nerve (pDNM-G) scaffold. Adapted with permission from Rao et al. [47] Copyright (2021) Elsevier. (**C**) Electrospinning process for preparing the conductive poly(ɛ-caprolactone) (PCL)/carbon nanotubes (CNTs) composite fiber with optimized alignment for promoting neural regeneration under electrical stimulation (ES). Adapted with permission from Zhang et al. [48] Copyright (2020) Wiley.

Microchannels, such as NGCs, are reported to have the ability to imitate the nerve fascicular (perineural) structure and inhibit axon dispersion, which could be designed by traditional production methods such as solvent casting [45]. The fabrication process of this strategy is extremely simple and accessible, which has made it a common method to produce NGCs in the past thirty years [46]. The procedure of solvent-casting includes dissolving the polymer in the solvent, filling the mold, and the evaporation of the solvent, which eventually results in a porous structure, as shown in Figure 3A. However, the drawbacks of this approach include the utilization of very hazardous solvents, inadequate pore interconnectivity, and uneven pore morphology. Therefore, it is critical to recognize these benefits as well as the limitations of the solvent casting in peripheral nerve regeneration.

To overcome the limitations mentioned above, combining solvent casting with other technologies to obtain a multistep process has been proven to be an effective method in the production of NGCs. Table 8 summarizes some common biofabrication methods of nerve guide conduits for peripheral nerve regeneration and demonstrates the application of solvent casting in the NGCs manufacturing process. For instance, Valentino et al. proposed and developed the poly(lactic-co-glycolic acid) (PLGA)/poly(d,l-lactic acid) (PDLLA) poly(ethylene glycol) 400 (PEG)-multichannel-based scaffolds (MCs) by the solvent casting and electrospinning method. A novel and promising microscale multichannel scaffold with safe, biodegradable, and biocompatible properties was achieved through this straightforward and efficient strategy, where the overall experimental results showed that the MCA platform was an appropriate option for creating a biomimetic environment at the injury site due to its aligned internal structure that promotes cell development [49]. Another polycaprolactone (PCL)/poly(lactic-co-glycolic acid) (PLGA) blended with different component ratios and PCL/PLGA composites containing 10% polypyrrole fibers (PPy) were synthesized by Ferreira et al. through the solvent casting technique. Their study illustrated that the proposed composite films, which were characterized by hydrophilic and porous surfaces, exhibited superior thermal stability and an appropriate degradation time for prospective use in peripheral nerve regeneration [50].

### 3.2. Freeze Drying

Another conventional method for constructing porous scaffolds for nerve regeneration is freeze drying, which enables the development of pores with sizes ranging from nanometers to micrometers [51]. Freeze drying, often known as freeze casting, is a prevalent technique for fabricating porous three-dimensional peripheral NGC structures. This method utilizes the vacuum sublimation of ice, transforming water into a solid status via chilling and then sublimating water molecules under vacuum conditions to produce porous materials [52,53]. As the pore structure is created by sublimating the solid ice crystals in a frozen condition, the freeze-drying technique preserves the physical three-dimensional shape of the nerve transplant. Under this situation, the unpredictable deformation of the peripheral nerve guidance conduit is prevented [53]. In general, there are three steps that comprise freeze drying, which are freezing, primary drying (ice sublimation), and secondary drying (removal of unfrozen water), respectively [54]. Due to its benefits of simple manufacturing process, low cost, and the ability to generate porous structures, the freeze-drying technique has been widely used in peripheral nerve regeneration, especially in the preparation of microchannels [55,56]. However, with this approach, the dimensions and configuration of the pores are relatively irregular in contrast to those produced by solvent casting, which is the main limitation to be considered by scientists during the fabrication process.

Table 8 concludes some recent research on freeze drying as the biofabrication method for NGCs manufacturing. For example, Rao et al. designed and investigated an innovative porcine sciatic nerve (pDNM-G) scaffold with longitudinally oriented microchannels, where the directed endoneurium-like structures with adjustable channel widths were reconstructed using unidirectional freeze drying, as illustrated in Figure 3B [47]. Furthermore, a novel graphene derivative named graphene oxide quantum dots (GOQDs) was introduced into the polycaprolactone (PCL) scaffolds through freeze drying by Yan et al. The experimental results demonstrated that GOQDs facilitated in the excretion of angiogenic agents by activating the ERK/CREB/VEGF pathway in macrophages, and the endothelial cells exhibited a strong capacity for tube production in vitro. The in vivo administration of the GOQD@PCL scaffold resulted in undetectable blood and organ toxicity but also enhanced motor, sensory, and electrophysiological recovery after peripheral nerve injuries (PNI), which provided a promising approach for possible translation into clinical practice and presented a viable path for nano-catalytic tissue regeneration [57].

### 3.3. Electrospinning

Electrospinning is a prevailing technique for fabricating microscale and nanoscale fibers. Due to the ability to imitate the native extracellular matrix (ECM), which could enhance cell–substrate interactions, electrospun fiber NGCs provide special benefits in peripheral nerve regeneration [58]. A high-voltage power supply, a grounded collector, a syringe pump, and a needle-equipped spinneret comprise the majority of the basic electrospinning apparatus [59,60]. The electrospinning device operates on a straightforward and explicit mechanism that is connected to the intricate electro-physical interaction between the surface of the polymer solution and the electrostatic force [60]. With this technique, random or directed fiber films can be produced, with the cross-space between the fibers generating a porous structure in the scaffolds. Through pore size control by adjusting the related parameters, the adaptable porous structure of electrospun NGCs can effectively avoid fibrous scar formation and enhance the migration of macromolecules and metabolic chemicals. More significantly, directional fibers can deliver direct physical signals that trigger axons to regenerate in a certain direction from the proximal to the distal terminal [61]. Nevertheless, the poor scalability and low reproducibility of this technology are the main factors limiting its development and clinical application.

Recent research has demonstrated that aligned fiber nerve conduits provide mechanical signals for axonal development and Schwann cell proliferation, and Table 8 demonstrates some recent research on electrospinning for nerve regeneration [62]. For instance, aligned electrospun poly-L-lactic acid (PLLA) nanofibers covered with decellularized peripheral nerve matrix were developed by Chen et al. and used in a dorsal root ganglion culture paradigm. Subsequently, accelerated axonal extension and remyelination were observed due to the topological guidance provided by the aligned electrospun fibers [63]. In addition, scaffold alignment and conductivity are two important variables affecting nerve healing effectiveness. Zhang et al. fabricated a series of conductive poly(ɛ-caprolactone) (PCL)/carbon nanotubes (CNTs) composite fibers with varying degrees of orientation through electrospinning at different rotational speeds (Figure 3C), which fully demonstrated the synergistic promotion mechanism of orientation morphology and electrical stimulation on nerve regeneration. The suggested conductive PCL/CNTs composite fiber with optimal matrix alignment significantly improved remyelination and axonal regeneration in vivo under electrical stimulation, indicating considerable promise as a tissue engineering strategy for addressing peripheral nerve injury [48].

### 3.4. Three-Dimensional Printing

Three-dimensional printing is the process of precisely stacking printing materials one layer at a time under computer control to develop the structure of any arbitrary object rapidly [64]. Through utilizing 3D model data, 3D printing has emerged as a dependable technique to fabricate biomaterials with intricate and accurate geometries, particularly for the construction of porous peripheral NGCs [65]. In contrast to conventional fabrication techniques as mentioned above, 3D printing can proficiently address the constraints of low surface porosity, yielding an extremely uniform and consistent three-dimensional porous structure. Furthermore, this technology can also address the issue of diminished mechanical performance in NGCs produced using traditional methods and electrospinning techniques [53].

**Figure 4:**
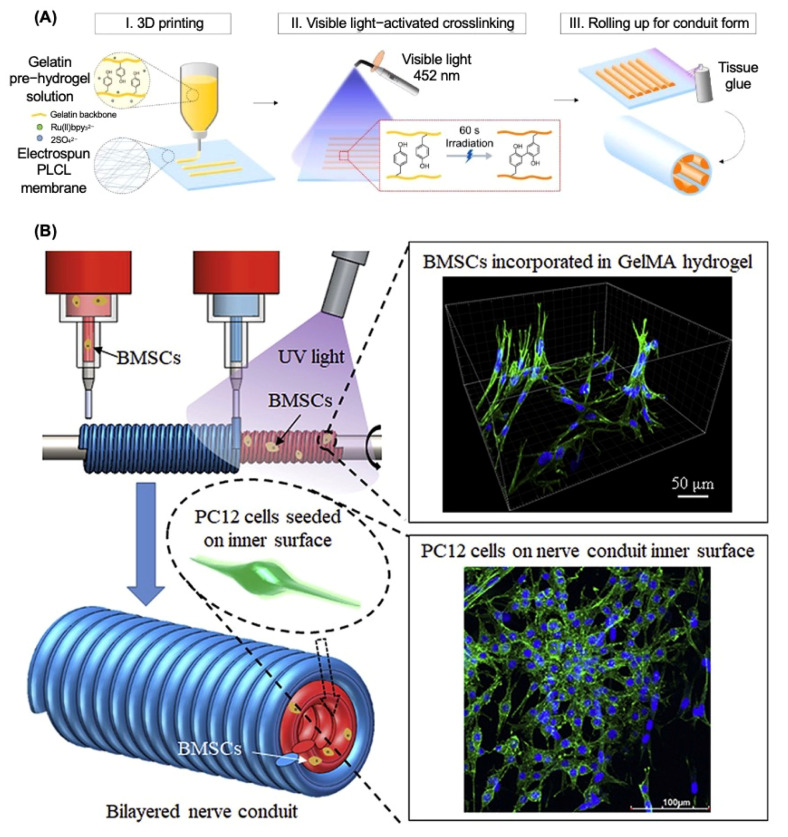
Additive manufacturing techniques for nerve guide conduit fabrication. (**A**) Three-dimensional printing with gelatin pre-hydrogel on poly(L-lactide-co-ε-caprolactone) (PLCL) membrane. Adapted with permission from Lee et al. [67] Copyright (2022) Elsevier. (**B**) Three-dimensional bioprinting with methacrylate-anhydride gelatin (GelMA) and bone marrow mesenchymal stem cells (BMSCs) for the inner layer of nerve guide conduits. Adapted with permission from Liu et al. [68] Copyright (2021) Elsevier.

Table 8 illustrates the summary of recent research on 3D printing as a manufacturing method for nerve regeneration. Li et al. developed a multiscale scaffold using high-resolution electrohydrodynamic (EHD) 3D printing, with suitable pore dimensions, an inductive construction, and improved mechanical reinforcement [66]. The experimental results demonstrated that the matrix of proposed NGCs had consistently dispersed holes that were homogeneous in size, facilitating superior permeability for cell infiltration, nutrition absorption, and waste elimination, which had the potential to mimic the microenvironment of the sciatic nerve and administer the therapy on the PNI rat model. Additionally, Lee et al. designed the inner-aligned gelatin hydrogel paths by combining 3D printing with the photocrosslinking system, which was introduced into the porous poly(lactide-co-ε-caprolactone) (PLCL) nerve guidance conduit to achieve multifunctional nerve regeneration (Figure 4A). The 3D-printed inner gelatin hydrogel and PLCL membrane functioned as a conducting instructing path and a flexible, non-collapsible epineurium, respectively, which have the ability to imitate the peripheral nerve’s fascicular morphology and successfully led to axonal regeneration with effective functional recovery [67].

In addition to ordinary 3D-printing strategies such as stereolithography (SLA) and digital light processing (DLP) techniques, 3D bioprinting has attracted tremendous attention in the field of peripheral nerve regeneration recently. The advancement of 3D bioprinting is significantly influenced by the bioink, which has been developed considerably in the past few years [17]. Briefly, 3D bioprinting incorporates live cells into bioinks and produces cell-loaded NGCs, and then, the regeneration capacity of NGCs can potentially be further improved by combining neurotrophic factors (NTFs) and medications with stem cells [69,70]. Recent breakthroughs in 3D bioprinting technology have enabled researchers to create innovative 3D scaffolds with intricate topologies to address the issues associated with dependable and precise neural regeneration. Among the neuroregenerative treatment strategies under investigation at present, 3D-bioprinted scaffolds provide the distinct benefit of considerable modifiability, enhancing their similarity to the natural biological structure of in vivo systems. The significant architectural resemblance between printed constructions and in vivo structures is believed to enhance the potential for repairing injured nerve tissues. However, the extremely high expense and manufacturing complications are the biggest obstacles to the development of 3D bioprinting in the field of neural regeneration [71]. It is essential to balance these benefits and limitations in the design and production of nerve conduits.

Scientists have tried to introduce different types of stem cells into the 3D-bioprinting system in recent years and Table 8 concludes some recent research on 3D bioprinting as the manufacturing method for NGCs fabrication. For example, a multi-nozzle additive-lathe 3D-bioprinting technique was used by Liu et al. to produce a completely integrated bilayered nerve conduit that incorporated bone marrow mesenchymal stem cells (BMSCs), as shown in Figure 4B [68]. The mechanical characteristics of the outer layer (poly(ethylene glycol) diacrylate) (PEGDA) were sufficient for skeletal support, while the inner layer (GelMA) was highly biocompatible and might provide a suitable microenvironment for nerve regeneration. The in vitro results indicated that this research provided an encouraging technique for the fabrication of bilayered nerve conduits with supporting cells for peripheral nerve regeneration. Moreover, Formaggio et al. proposed a novel 3D-bioprinted fibroblasts/dorsal root ganglion (DRG) co-culture construct to investigate and clarify peripheral nervous system (PNS) wiring and regeneration mechanisms in both physiological and pathological conditions, which established the foundation for developing individualized treatments for neuropathic pain and sensory dysfunction, enhancing both basic neuroscience and translational medicine [72].

**Table 8:** Manufacturing methods of nerve guide conduits for peripheral nerve regeneration.

Study/Researcher	Materials/Cells Application	Fabrication Method	Specific Outcomes
Valentino et al. [49]	PLGA, PDLLA, PEG	Solvent casting; electrospinning	The microscale multichannel conduit with safe, biodegradable, and biocompatible properties was investigated via multiple techniques combination
Ferreira et al. [50]	PCL, PLGA, PPy fibers	Solvent casting	The designed conduit illustrated the superior thermal stability and an appropriate degradation time for prospective use in peripheral nerve regeneration
Rao et al. [47]	pDNM-G	Freeze drying	The bioactive pDNM-G NGCs promoted the regulated release of neurotrophic factors and efficient incorporation of topological cues, potentially facilitating future therapeutic interventions for peripheral nerve injuries
Yan et al. [57]	GOQDs, PCL	Freeze drying	The proposed GOQD@PCL scaffolds enhanced intraneural vascularization and enhanced the microstructural repair of peripheral nerves while contributing to minimal fibrotic capsule development
Chen et al. [63]	PPLLA, pDNM	Electrospinning	The electrospun cell culture platform illustrated the promotion of Schwann cell–neurite interactions
Zhang et al. [48]	PCL, CNTs	Electrospinning	The designed PCL/CNTs NGCs were advantageous for the directed development of neural cells in vitro and the repair of damaged sciatic nerves in vivo
Li et al. [66]	PCL, NCSCs	EHD 3D printing	The multifunctional 3D-printed scaffold facilitates directional development and myelination, resulting in the regrowth of myelinated nerves with enhanced density and the absence of visible scaffolds after half a year
Lee et al. [67]	Gelatin, PLCL	3D printing	The designed 3D-printed PLCL/gelatin conduit exhibited effective axonal regeneration and remyelination capabilities and promoted functional recovery
Liu et al. [68]	PEGDA, GelMA, BMSCs	3D bioprinting	The proliferation and neurite outgrowth of PC12 cells cultured on bilayered BMSCs-encapsulated nerve conduits were dramatically enhanced, which suggested significant potential in peripheral nerve regeneration
Formaggio et al. [72]	Sodium alginate, gelatin, fibrinogen, fibroblasts, DRG	3D bioprinting	The designed 3D-bioprinted fibroblasts/DRG co-culture construct provided a comprehensive in vitro foundation for investigating sensory system reinnervation

## 4. Discussion

Nerve repair presents a considerable challenge in clinical practice, mainly due to the limitations of conventional methods like autografting, which can lead to donor site morbidity and variability in recovery outcomes. As a result, NGCs have been developed as an alternative, prompting research into various biomaterials and bioengineering techniques to boost nerve regeneration capabilities. Contemporary conduits now incorporate bioactive substances—such as growth factors and components of the extracellular matrix—to create an optimal environment for nerve healing and axonal development. The FDA has approved several NGCs, including Neuromatrix^®^ (collagen). However, statistics show that the use of these FDA-approved conduits remains low, often falling below 10% of nerve repair surgeries, primarily due to challenges like high costs, complex manufacturing processes, and less effective performance in bridging larger nerve gaps compared to autografts.

This literature review encompasses some of the most recent studies on popular biomaterials used in nerve repair and regeneration, particularly those related to nerve guidance conduits and innovative bioengineering techniques. Researchers have employed sophisticated designs and explored combinations and variations of biomaterials to enhance mechanical properties. A systematic review comparing the effectiveness of autografts and NGCs found that autografts offer better functional recovery and regeneration. While NGCs appear to facilitate nerve regeneration at the cellular level, the functional recovery outcomes often do not exceed those achieved through traditional methods, such as autografts, highlighting the hurdles that must be overcome with nerve conduits [73].

Recent advancements in nerve repair, particularly through the development of NGCs, have yet to be widely adopted in clinical practice due to persistent challenges in achieving effective therapeutic outcomes. Research into various biomaterials and innovative bioengineering techniques has led to diverse NGC designs that often incorporate bioactive elements such as growth factors or utilize combinations of materials, including collagen and chitosan, in composite conduit setups. These efforts aim to create conduits that not only facilitate nerve regeneration physically but also foster a supportive environment for cellular growth and axonal development. However, despite encouraging findings from experimental studies, the translation of these innovations into clinical settings has been a slow and often frustrating endeavor.

The use of stem cells within nerve guidance conduits for peripheral nerve repair presents significant challenges that limit their clinical application. Ethical concerns surrounding the use of human stem cells complicate their application, as the collection of these cells typically requires invasive procedures. Additionally, the process of obtaining autologous cells can lead to inconsistencies and treatment delays—critical issues in emergencies that require swift action. Moreover, ensuring cell viability and functionality during their integration into conduits adds further complexity. The variable conditions surrounding a peripheral nerve injury may affect the viability and effectiveness of the incorporated cells and growth factors, which may struggle to operate under physiological stress. Furthermore, integrating multiple growth factors or bioactive agents, along with the necessary technologies to construct advanced cell-seeded conduits, could be impractical for clinical use due to high labor requirements and associated costs.

Future directions for enhancing artificial NGCs should focus on overcoming current limitations related to their effectiveness and clinical application. Improving the mechanical properties of conduits by integrating novel biomaterials, such as biodegradable polymers and composite materials, can enhance their performance in bridging larger nerve gaps. Furthermore, optimizing the release profiles of bioactive agents like growth factors within the conduits may achieve a more sustained and localized effect on nerve regeneration. Using advanced manufacturing methods, such as 3D printing, can enable the production of patient-specific conduits customized to individual anatomical and injury characteristics. Ultimately, a multidisciplinary approach that combines bioengineering, pharmacology, and regenerative medicine will be essential in developing NGCs that not only support nerve repair but also restore function to levels similar to those of traditional autografts.

Given these challenges, one might reconsider the current focus on NGC development and explore alternative strategies to improve nerve healing. From a clinical and surgical perspective, tensionless nerve repair or autografts remain the most dependable methods for nerve regeneration, highlighting the importance of recognizing the innate healing abilities of peripheral nerves. Focusing on pharmaceutical innovations that enhance these healing processes could potentially reduce some of the limitations of conduit technology. Pharmacological approaches that support neuroprotection, boost cellular proliferation, and stimulate extracellular matrix production may help optimize this natural healing capacity, thereby easing the difficulties associated with artificial conduits. Furthermore, investigating combinations of growth factors and signaling molecules to activate local Schwann cell populations may create more favorable conditions for nerve regeneration. Researching small-molecule drugs that influence pathways related to nerve repair and inflammation might offer a less resource-intensive alternative for achieving quicker therapeutic outcomes compared to the lengthy development timelines required for new biomaterial constructs.

## 5. Conclusions

This review highlights recent advancements in NGCs, focusing on biodegradable materials, including collagen and chitosan, as well as non-biodegradable options such as PTFE. Each material offers distinct advantages and disadvantages, which drive ongoing research. Innovators are developing conduits with complex designs that can incorporate bioactive agents, including growth factors, to enhance nerve regeneration results. However, reaching a consensus on the best conduit design or material that consistently yields superior outcomes in clinical environments remains difficult, as many positive results are largely confined to experimental phases and have not yet been successfully applied in surgery. These encouraging findings often fall short of practical clinical significance, underscoring the need to reassess foundational approaches to nerve repair.

## Figures and Tables

**Table 1:** Summary of recent research on collagen as a biomaterial for nerve regeneration.

Study/Researcher	Collagen Application	Additional Components	Specific Outcomes
Kim et al. [18]	NGC combining PRP with collagen	PRP	Increased Schwann cells proliferation and migration; enhanced vocal cord mobility; reduced muscle atrophy; faster axon regrowth; increased neurotrophic factors
Takeya et al. [19]	Hydrogel NGC combining chitosan and collagen	Schwann cells encapsulated	Schwann cells-encapsulated chitosan–collagen hydrogel nerve conduits (CCNs) showed improved motor functional recovery, axonal regeneration, and myelination

**Table 2:** Summary of recent research on chitosan as a biomaterial for nerve regeneration.

Study/Researcher	Chitosan Application	Additional Components	Specific Outcomes	Notes
Deng et al. [23]	Chitosan-based nerve conduit via electrodeposition	Conductive hydrogel, DHF (drug)	Enhanced myelin sheath and axon regeneration; recovery of gastrocnemius function improved; nerve repair effectiveness comparable to autologous transplantation	Bilayer composite conduit allows controlled drug release and mechanical stability
Zhu et al. [15]	Chitosan microspheres as 3D porous microcarriers	Adipose-derived stem cells, ADSCs	Improved nerve regeneration in rat model; increased ADSC proliferation; secretion of VEGF and TGF-β enhanced nerve repair	3D porous framework enhances cell retention and functionality compared to 2D cultures
Li et al. [24]	Lysine-modified chitosan porous scaffold	Matrilin-2, collagen conduit	Promoted Schwann cell migration and axonal growth; improved mechanical strength and biodegradation rate	Chitosan’s lower Young’s modulus benefits flexibility and reduces failure; interaction with collagen critical for optimal regeneration

**Table 3:** Summary of recent research on GelMA as a biomaterial for nerve regeneration.

Study/Researcher	GelMA Application	Additional Components	Specific Outcomes	Notes
Cai et al. [25]	Conductive GelMA-based hydrogels	Growth factor gradients, graphene, silk fibroin (double-network hydrogel)	Improved mechanical properties; supported Schwann cell proliferation and viability; targeted therapeutic delivery; enhanced functional recovery in diabetic mice	GelMA/silk fibroin hydrogel has rapid setting, mechanical support, biocompatibility, and sustained growth factor delivery
Xu et al. [20]	GelMA gels infused in electrospun PCL conduits	CNTF, IGF-1	Significant nerve regeneration and functional recovery in rat sciatic nerve injury; prolonged release of growth factors; augmented Schwann cell proliferation and axonal growth	GelMA-CNTF/IGF-1 composite hydrogel enables controlled neurotrophic factor release

**Table 4:** Summary of recent research on biodegradable synthetic polymers for nerve regeneration.

Study/Researcher	Synthetic Polymer Application	Additional Components	Specific Outcomes	Notes
Kusuhara et al. [34]	PGA	Collagen	Enhanced sensory recovery and reduced transplant rejection	Bridging nerve gaps
Cicero et al. [35]	PBS	Electrospinning technologies	Improved nerve regeneration in living organisms	Superior biocompatibility and decreased inflammation
Nazeri et al. [36]	PLGA	Laminin, carbon nanotube (CNTs) and poly(dopamine) (PD)	Promoted neurite growth and cell interactions	PD coating for surface modification

**Table 5:** Summary of recent research on PTFE as a biomaterial for nerve regeneration.

Study/Researcher	PTFE Application	Additional Components	Specific Outcomes	Notes
Labroo et al. [40]	PTFE nerve-guiding conduits	Neuroprotective substances: GDNF, FK506, micro-grooved surfaces, and porous features	Enhanced axon growth and functional recovery; facilitated neuronal cell adhesion and robust axon extension	Hole-based drug delivery system allows controlled release of growth factors; biocompatible; customizable drug types and dosages
Kahraman et al. [41]	PTFE combined with hydrophilic materials	PEG, collagen	Improved cell adherence and biocompatibility; composite conduits support cell movement and growth	Creating composite conduits that support structure and cellular function

**Table 6:** Summary of recent research on silicone as a biomaterial for nerve regeneration.

Study/Researcher	Silicone Application	Additional Components	Specific Outcomes	Notes
Xie et al. [21]	Silk sericin/silicone nerve guidance conduit	Silk sericin	Enhanced regeneration of transected sciatic nerves; improved muscle weight recovery and nerve regeneration	Combination leverages biocompatibility of silk and mechanical strength of silicone
Velichanskaya et al. [42]	Silicone conduit filled with mixture	ADSCs and collagen	Promoted nerve regeneration with satisfactory degrees of regeneration	Demonstrates benefits of combining silicone with living cell therapies

**Table 7:** Summary of recent research on smart polymers as biomaterials for nerve regeneration.

Study/Researcher	Polymer Application	Additional Components	Specific Outcomes	Notes
Rahman et al. [43]	Electrically conductive smart polymers in NGCs	Electrical stimulation	Improved functional recovery and histological nerve regeneration	Facilitates electrical stimulation during active proliferation state
Jaswal et al. [22]	Smart polymers combined with conductive nanoparticles	RGO, AuNPs	Enhanced mechanical and electrical properties of polymers; improved axonal growth and guidance	Nanocomposites maximize healing efficacy and expand conduit functionality
